# Using the objective structured clinical examinations in undergraduate midwifery students

**Published:** 2013-03-25

**Authors:** MA Delavar, H Salmalian, M Faramarzi, H Pasha, A Bakhtiari, M Nikpour, FM Ledari

**Affiliations:** * Fatemezahra Infertility and Reproductive Health Research Center, Department of Midwifery, Babol University of Medical Sciences, Babol, Iran; ** Department of Midwifery, Babol University of Medical Sciences, Babol, Iran

**Keywords:** midwifery education program, educational measurement, objective assessment, program evaluation

## Abstract

The Objective Structured Clinical Examination (OSCE) has been considered a modern type of examination for the assessment of clinical skills within nurse education, but it has been rarely applied in the teaching of midwifery. The aim of the present study was to assess the use of the OSCE as a tool to evaluate the abilities of undergraduate midwifery students and to compare the perspectives of the students regarding the OSCE and traditional examination. Fifty-two midwifery students participated in the study. The export trainer evaluated the internal consistency of the OSCE stations and it was tested by using Cronbach’s alpha. Successive groups of students completed a self-administered questionnaire immediately after the final examination. The students’ perspective regarding the traditional final examination ranked as unsatisfactory by more than two thirds of the students, while, the students’ perspective regarding the OSCE system was ranked as very satisfactory to satisfactory by more than half of the students (p=0.001). There was a significant difference in the students’ perspective between the OSCE system and the traditional final examination among the students (49.8±18.3 vs 25.3±18.1) (p=0.001). A significant difference was found in being credible (p=0.0001), consistent/reliable (p=0.001), enhances teaching level (p=0.011), and measures the course category (p=0.008) between two methods of the final examination. Around half of the students expressed their opinion that the OSCE test was a stressful assessment. Overall, students’ evaluation of the OSCE was remarkably encouraging. To this end, we recommend the consideration of the validity and reliability of the process for undergraduate midwifery students.

## Introduction

In the Iranian system, the increasing number of midwifery students enrolled in the nursing and midwifery faculties, who trained as midwife without being nurses first (direct entry midwives ). Midwifery education takes at least 4 calendar years of study for both classroom instructions and clinical posting in a Medical University. After finishing their remaining studies, midwifery students must take in the clinical final examination. The final exam is the most important examination for midwifery students during 4 calendar years of the study. The successful students at the final examination conducted by nursing and midwifery faculty are designated as graduated of midwife with bachelor’s degree. The final examination includes short cases, long cases and oral examination in four fields; delivery room, clinical gynecological unit, prenatal care unit and mothers and children health. The traditional clinical final examination is several problems and is not standardized to assess clinical competency and clinical reasoning skills. The teacher often questions only regarding his final conclusion and gives summation score and does not observe history taking, physical examination, diagnostic reasoning and management of the student [**1,2**]. In addition, there is a limited resource from the clinical sites. Thus, practice on patient may not be available for students. As the number of midwifery students is increased, seemingly, this traditional final examination cannot be used to measure the clinical skills. However, one study in Mashhad (Iran) showed that the assessment instruments of the traditional clinical final examination for undergraduate midwifery students was valid and reliable in the assessment of the students at their educational career [**3**]. 

 The Objective Structured Clinical Examination (OSCE) had been established as part of the repertoire in the clinical assessment skill in a standardized content for over three decades [**1,4-5**]. Medical faculties in Iran use OSCE for testing medical, dental students’ outcome. However, in Iran, there is no available evidence for using OSCE in midwifery education. Since 1985, OSCE has been adopted for the assessment of the nursing skills in Canada [**6**]. Researchers have shown that OSCE is a valid and reliable assessment to evaluate the student’s clinical performance [**3,7-11**]. Many studies showed that the OSCE can be used most effectively in nurse undergraduate curricula to assess safe practice in term of performance of skills [**12,15**]. In 2000, for first time, Hong Kong midwives council used the OSCE as a component of the Certification examination on midwife in the school of midwifery, adapted a new assessment system and reported that the OSCE is a gold standard assessment to assess the clinical competency of midwives. Therefore, we designed the final OSCE method to assess midwifery students in final examination and compared perspectives of undergraduate midwifery students regarding the final OSCE and traditional final examination.

## Methods

In this cross-sectional study, 52 midwifery students participated in the study. All the participants were healthy without any history of severe stress. The data were collected by using a special questionnaire; data of age, mother’s and father’s educational level, father’s and mother’s age, and marital status.

 Two groups of midwifery students from the Faculty of Midwifery of Babol University of Medical Sciences participated in the process during the final examination, one group in November 2010 and another one in July 2011. The first trial was conducted in November in the delivery room, clinical gynecology unit, prenatal care unit and mothers and children health; 20 students were evaluated by using the traditional method of evaluation in July 2011, 32 students were evaluated by using the OSCE method. Students of the groups had two briefing sessions before the OSCE, and included an orientation about the examination process. In the first step of the study, training of trainers was done for OSCE. Ethical review of the study project was obtained in several workshops. They were held for clinical instructors and faculty members by the Educational Development Center of Babol University of Medical Sciences (EDC) to raise the awareness of OSCE. The panel examiner was selected among the export trainer who held the design and conduct of the OSCE. 16 checklists to assess the student’s clinical competence in the OSCE were prepared by the clinical instructors and faculty members. The checklists were mostly developed by the panel examiner. Face and content validity of each check list and the student’s perspective questionnaire was the coefficient test, the reliability of the student’s perspective questionnaire was of 0.81 and the reliability of the checklists applied in station 1 to 16 were between 0.67 and 0.86.

 The standard patients were selected among the expert midwives. They were trained so that the standard patient did not change in each station during the examination. The time of each station was of six minutes. One minute was given between stations to facilitate change and the reading of the instructions. All the students completed the circuit in a period of almost two hours. The aim of each station was to test a particular clinical competence. All the necessary instruments and instructions for the students’ performed requested skills and their performances were assessed according to the criterion reference for each station. The criterion based scoring was used with each checklist item. The observer filled the checklist based on the related rating scale, each checklist item score being 0 (omitted incorrect or inadequate) or 1-2 (correct or adequate). 

 In several questions they were asked to assess the students’ perspectives regarding the OSCE and traditional final examination score on the scale was of 0.0 (strongly disagree) to 4.0 (strongly agree). For each subject, the score of subject question was converted into percentage to represent the students’ perspective. A score of at least 30% was considered as a “satisfactory" perspective regarding the method of final examination, a score below 30% was considered as “unsatisfactory" and above 50% as “very satisfactory". 

 All the analyzes were performed with SPSS (version 16.0). The descriptive statistics were used to describe the mean scores and proportion. To compare the students’ perspective regarding two methods of final examination, t test and qui-square test were done. All the analyses were performed by using two-tailed hypotheses testing the level of significance set at 0.05.

## Results

COf 52 midwifery students, all were women; the mean ± SD age of the study participants was 23.1±0.7 years. Twenty students (38.5%) were married. The mean education of the students’ father and mother were 11.6±4.5 and 9.9±4.0 years, respectively. Also the mean age of the students’ father and mother was 52.3±3.5 and 47.8±4.9 years, respectively. Half (50.0%) of the students reported that they do not get enough sleep and had stress of final examination. The main cause of stress (53.8%) was reported to be the examination system itself.

**Table 1** shows the students’ perspective regarding the traditional final examination. It was ranked as unsatisfactory by more than two thirds of the students with a mean score of the students’ perspective of 25.3±18.1. While the students’ perspective regarding the OSCE system was ranked as very satisfactory to satisfactory by more than half of the midwifery students with a mean score of the students’ perspective of 49.8±18.3 (**Table 2**).

**Table 1:** Distribution of the students’ perspectives regarding the traditional final examination (N=20)

	Very satisfactory n(%)	Satisfactory n(%)	Unsatisfactory n(%)
Is credible	1(5.0)	2(10.0)	17(85.0)
Is consistent/reliable	2(10.0)	2(10.0)	16(80.0)
Enhances teaching level	3(15.0)	4(20.0)	13(65.0)
Measures the course category	4(20.0)	2(10.0)	14(70.0)
Mean ± SD		25.3±18.1	

**Table 2:** Distribution of the students’ perspectives regarding the OSCE system (N=32)

	Very satisfactory n(%)	Satisfactory n(%)	Unsatisfactory n(%)
Is credible	12(37.5)	5(15.6)	15(46.9)
Is consistent/reliable	10(31.2)	7(21.9)	15(46.9)
Enhances teaching level	11(34.4)	7(21.9)	14(43.8)
Measures the course category	11(34.4)	9(28.1)	12(37.5)
Mean ± SD		49.8±18.3	

 There was a significant difference in the students’ perspective between the OSCE system and traditional final examination among midwifery students (**Table 3**).

**Table 3:** Comparison of the OSCE versus traditional final examination among undergraduate midwifery students

Score category	OSCE (N=32) n(%)	Traditional final examination (N=20) n(%)	p-valve
Unsatisfactory	6(18.8)	11(55.0)	0.001
Satisfactory	13(40.6)	9(45.0)	
Very satisfactory	13(40.6)	0(0.0)	

**Table 4** illustrates the comparison between the mean students’ perspective and the students who underwent OSCE and traditional final examination. The significant difference was found in being credible (p=0.0001), consistent/reliable (p=0.001), enhancing the teaching level (p=0.011), and measuring the course category (p=0.008) between the two methods of final examination. In addition, students who underwent the final OSCE reported that the final OSCE is more stressful (p=0.0001) and is a more fearful form of assessment (p=0.0001) compared with the traditional final examination.

**Table 4:** Comparison of the students’ mean perspectives of the OSCE versus the traditional final examination among undergraduate midwifery students

	OSCE mean ±SD	Traditional final examination mean ±SD	P-value
Is credible	19.2±25.8	21.3±29.3	0.0001
Is consistent/reliable	22.5±2	22.5±28.8	0.007
Enhances teaching level	49.2±27.3	28.15±27.7	0.001
Measures the course category	51.3±26.5	28.8±34.7	0.008
Is stressful	53.1±29.6	86.3±23.6	0.0001
Is fearful	42.2±29.4	82.5±28.2	0.0001

## Discussion

In this study, around half of students expressed their opinion that the OSCE test was a stressful assessment. Moreover, 40 percent of the students felt that the final OSCE process was fair and they reported that the time allocated to each station was not satisfactory. These results were consistent with many studies that a considerable percentage of students viewed OSCE as a stressful assessment [**9,15-17**]. A possible explanation for the perception is the new experience among students with increased anxiety. The anxiety is related to the students’ stress [**18,20**].

 In relation to the students’ perspective regarding the OSCE system, more than half of the students indicated that the OSCE system is credible, consistent, reliable, enhances the teaching level and also measures the course category. The findings were somewhat similar to the view of students at Cario and Ain Shams Universities of Egypt [**21**]. However, the midwifery students’ perspective was positive in some area but seemingly, the OSCE had few limitations including insufficient time for each station that, in some studies, was one of the students’ complains [**19-22**] and stressful.

 Student perception of the OSCE however may have been influenced by anxiety, in adequate preparation for the examination, and lack of confidence was associated with a new assessment. Also the students completed the questionnaire immediately after the final examination, hence the students’ stress and fatigue should be taken into consideration.

## Conclusions

Our results showed that a new assessment tool in the final examination is needed for midwifery curricula. The traditional final examination is not a suitable tool in the evaluation of the clinical competence in midwifery. In this study, the students’ evaluation of OSCE was encouraging. We would like to recommend the OSCE; it could be used to evaluate the skills of undergraduate in the future final examination. It requires the consideration of validity and reliability of the process for undergraduate midwifery students. 

 Acknowledgment

 We wish to thank Dr. Iman Jahanian, Maryam Ghaemi and Kolsoum Vajedi for the professional advice and permission to use the OSCE in this study. We also express our gratitude to the participating students and lecturers in the midwifery department who contributed to the implementation of the OSCE in the department. We also appreciated the standard patients, observed while including Sayed Zahra Banihoseini, Hamide Abdollazade and all the students who participated in giving their feedback.

 Competing interests 

 The authors declare that there is no conflict of interest.
